# 拓扑替康用于小细胞肺癌维持/巩固治疗和挽救治疗的疗效比较

**DOI:** 10.3779/j.issn.1009-3419.2010.03.05

**Published:** 2010-03-20

**Authors:** 玮 蒋, 阳 张, 洪云 赵, 光川 徐, 立平 林, 媛媛 赵, 聪 薛, 力 张

**Affiliations:** 510060 广州，中山大学肿瘤防治中心，华南肿瘤学国家重点实验室 State Key Laboratory of Oncology in Southern China, Sun Yat-Sen University Cancer Center, Guangzhou 510060, China

**Keywords:** 肺肿瘤, 拓扑替康, 化疗, Lung neoplasms, Topotecan, Drug therapy

## Abstract

**背景与目的:**

小细胞肺癌（small cell lung cancer, SCLC）作为治疗有效率和复发率都很高的疾病，如何减少其复发或延长缓解时间、改善总生存，一直是目前亟待解决的问题。本研究旨在探索拓扑替康（topotecan, TPT）用于SCLC维持/巩固治疗的临床获益。

**方法:**

回顾性分析53例SCLC患者的治疗结果，按治疗方法分为两组：一线化疗取得缓解或稳定后用TPT维持或巩固的维持/巩固组和TPT用于二线或二线以后的挽救治疗组。用*Log-rank*法对总生存差别进行单因素分析比较，并行多因素*Cox*回归分析。

**结果:**

维持/巩固组29例，挽救化疗组24例，客观有效率分别为51.7%和41.7%，中位生存时间分别为20个月和27个月（*P*=0.89）。*Cox*回归分析显示仅性别和分期是有统计学意义的预后因素。

**结论:**

将TPT提前用于一线取得缓解或稳定的SCLC维持/巩固治疗能提高有效率，但未见生存优势。

## Abstract (en)

**Background and objective:**

How to prolong progression free survival (PFS) and overall survival (OS) of patients with small cell lung cancer (SCLC) has been one of the hottest issues. We retrospectively reviewed our data to compare the survival of immediate with delayed topotecan after first-line therapy in SCLC.

**Methods:**

In our retrospective study, 53 patients with SCLC were divided into two groups as follow: patients receiving topotecan-containing regimen as maintenance/ consolidation (maintenance/consolidation chemotherapy group) and salvage chemotherapy (salvage chemotherapy group). The *Log-rank* test was used to assess the difference in OS between two groups. *Cox* regression model was used for the multivari- able analysis of independent prognostic factors.

**Results:**

Twenty-nine patients received topotecan as maintenance/consolida- tion treatment, whereas 24 patients salvage chemotherapy. The response rates were 51.7% and 41.7%, respectively. The median survival time were 20 months and 27 months respectively (*P*=0.89). Multivariate *Cox* regression analyses identified sex and stage as independent prognostic factors.

**Conclusion:**

Efficacy of first-line therapy was improved by topotecan maintenance/ consolidation treatment, which did not result in any significant survival benefits in SCLC.

小细胞肺癌（small cell lung cancer, SCLC）是恶性程度最高的肿瘤之一，预后较差，2年生存率低于10%^[1]^。按美国退伍军人协会（Veterans Administration Lung Study Group, VALG）分期，局限期SCLC定义为半胸即包括肺、肺门、纵隔和/或同侧锁骨上淋巴结，广泛期SCLC为病变超过局限期范围。多数SCLC诊断时已为广泛期，局限期仅占1/3^[2]^。SCLC治疗较为敏感，局限期首选同期化放疗，广泛期以化疗为主。广泛期推荐的一线化疗方案依托泊苷+顺铂（EP）有效率约40%-70%，局限期EP方案联合放疗有效率可达60%-90%^[3]^。SCLC近期疗效较好，但远期结果较差，很多患者在缓解后不久出现复发进展，且复发后治疗的敏感性较差，大部分患者2年内死亡。目前报道^[4]^SCLC中位生存时间（median survival time, MST）局限期为18个月-20个月，广泛期仅8个月-12个月。局限期SCLC的5年生存率为15%-25%，而广泛期小于5%^[5]^。因此，如何减少肿瘤复发或延长缓解时间、改善患者总生存，一直是SCLC的研究热点。*meta*分析^[6]^显示维持/巩固治疗可提高1年及2年生存率和无进展生存（progression free survival, PFS），但该研究一线及维持/巩固化疗方案并不统一，何种方案更为有效也没有一致的结论。

拓扑替康是拓扑异构酶Ⅰ抑制剂，对SCLC有确切疗效。研究^[7]^显示单药拓扑替康与CAV方案（环磷酰胺+阿霉素+长春新碱）用于一线治疗结束2个月-3个月以上复发或进展的SCLC有相似的有效率和总生存，且拓扑替康组疾病相关症状改善率更高。据此，拓扑替康被批准用于一线化疗结束后2个月-3个月进展的SCLC。

拓扑替康是单药治疗SCLC的有效药物，且患者耐受性良好，无累积毒性，是维持/巩固治疗理想的候选药物。Schiller等^[8]^报道一线化疗取得肿瘤控制的SCLC接受拓扑替康维持较观察组PFS延长，但总生存的延长无统计学意义。为此，我们进行了回顾性分析，探讨将二线的拓扑替康方案提前用于SCLC的维持/巩固治疗是否有生存获益。

## 1 资料与方法

### 1.1 病例资料

2000年4月-2003年12月中山大学肿瘤防治中心收治的部分SCLC病例，24例接受含拓扑替康方案挽救化疗，29例接受含拓扑替康方案维持/巩固化疗。所有病例经细胞学或组织学确诊，治疗前后均进行了详尽的影像学检查。按照VALG分期分为局限期和广泛期。化疗前常规行血常规、肝肾功能、心电图检查及体力状况（ECOG performance status, PS）评价。局限期患者一线化疗时均同期行放疗。

### 1.2 化疗方案

所有患者均接受单药拓扑替康（1.25 mg/ m^2^-1.5 mg/m^2^，d1-d4或d1-d5）或拓扑替康（1.25 mg/ m^2^-1.5 mg/m^2^，d1-d4或d1-d5）+顺铂（20 mg/m^2^，d1-d5；或75 mg/m^2^-80 mg/m^2^，d1）治疗，均为3周方案。

### 1.3 客观疗效和毒性的评价

按RECIST标准（Response Evaluation Criteria in Solid Tumors）进行疗效评价，分为完全缓解（complete response, CR）、部分缓解（partial response, PR）、稳定（stable disease, SD）和进展（progres- sive disease, PD）。维持治疗的疗效判断以维持治疗开始前的最近一次影像学评价作为基线值，按RECIST标准进行疗效评价（如一线治疗取得CR，则维持治疗疗效评价为CR）。有效率定义为CR+PR占总病例数的比例。总生存（over survival, OS）定义为患者开始治疗至死亡时间或末次随访时间。毒性反应根据美国国立癌症研究所制定的通用毒性评价标准（Common Toxicity Criteria, CTC）3.0版进行分级。

### 1.4 统计学分析

采用SPSS 16.0统计软件进行分析，采用*t*检验、*χ*^2^检验、*Log-rank*检验及*Cox*逐步回归法进行分析。*P* < 0.05为差异有统计学意义。

## 2 结果

### 2.1 病例资料结果

53例接受拓扑替康挽救或巩固/维持治疗的SCLC的临床特征情况见表 1。中位年龄为53岁（40岁-78岁），男性43例，占病例总数的81.1%，ECOG PS评分0分-2分有52例（98.1%），局限期35例（66.0%）。24例在疾病进展后接受含拓扑替康方案挽救治疗，29例一线治疗肿瘤控制后改含拓扑替康方案维持/巩固。末次随访时间为2009年6月。

**1:** 患者临床特征 Patient characteristics

Characteristics	Maintenance/Consolidation chemotherapy (*n*=29)	Salvage chemotherapy (*n*=24)	*P*
Age, yr, median (range)	53 (40-70)	57 (41-78)	0.19
Sex (*n*/%)			0.74
Male	24 (82.8%)	19 (72.9%)	
Female	5 (17.2%)	5 (20.8%)	
Age group (*n*/%)			0.79
≥65	26 (89.7%)	20 (83.3%)	
< 65	3 (10.3%)	4 (16.7%)	
Stage (*n*/%)			0.28
Limit stage	21 (72.4%)	14 (58.3%)	
Extensive stage	8 (27.6%)	10 (41.7%)	
ECOG PS (*n*/%)			0.35
0	0 (0.0%)	1 (4.2%)	
1	25 (86.2%)	19 (79.2%)	
2	4 (13.8%)	3 (12.5%)	
3	0 (0.0%)	1 (4.2%)	
No. of metastatic sites (*n*/%)			0.07
0	21 (72.4%)	14 (58.3%)	
1	8 (27.6%)	7 (29.2%)	
2	0 (0.0%)	3 (12.5%)	
Elevated ALP level (*n*/%)	4 (13.8%)	3 (12.5%)	0.89
Decreased albumin (*n*/%)	2 (7.0%)	5 (20.8%)	0.28
Elevated LDH level (*n*/%)	9 (31.0%)	9 (37.5%)	0.62
ALP: alkaline phosphatase; LDH: lactate dehydrogenase; PS: performance status.			

### 2.2 拓扑替康挽救治疗结果

24例患者疾病复发或进展后接受了含拓扑替康方案挽救治疗（表 2）。诊断时局限期患者14例（58.3%），广泛期10例（41.7%）。大部分患者一线化疗方案同时包含了足叶乙甙和铂类（23例，95.8%）。末次一线治疗时间距复发转移 < 3个月（耐药复发）10例（41.7%）， > 3个月（敏感复发）的患者14例（58.3%）。挽救治疗拓扑替康单药14例（58.3%），与顺铂联合治疗10例（41.7%）。中位化疗周期数为2。拓扑替康作为二线治疗15例（62.5%），三线及三线以后的治疗9例（37.5%）。治疗后PR占41.7%（10/24），SD占12.5%（3/24），PD占25.0%（6/24），5例（5/24, 20.8%）缺乏疗效评价资料。

**2:** 治疗方案及疗效 Therapeutic regimen, scheme and outcome

Regimen, scheme and outcome	Maintenance/Consolidation chemotherapy (*n*=29)	Salvage chemotherapy (*n*=24)	*P*
Cycle, median (range)	2 (1-4)	2 (1-11)	
Etoposide and platinum-based first-line regimen (*n*/%)	28 (96.4%)	23 (95.8%)	0.71
Topotecan treatment (*n*/%)			0.06
Topotecan	9 (31.0%)	14 (58.3%)	
Topotecan plus cisplatin	20 (69.0%)	10 (41.7%)	
Efficacy (*n*/%)			< 0.001
CR	8 (27.6%)	0	
PR	7 (24.1%)	10 (41.7%)	
SD	5 (17.2%)	3 (12.5%)	
PD	3 (10.3%)	6 (25.0%)	
Unkown	6 (20.7%)	5 (20.8%)	
Survival			
1-year	86%	91%	0.78
2-year	47%	62%	0.63
5-year	19%	5%	0.79
MST, months	20	27	0.89
CR: complete response; PR: partial response; SD: stable disease; PD: progressive disease; MST: median survival time.			

### 2.3 拓扑替康的维持/巩固治疗结果

29例一线治疗取得CR、PR或SD的患者在治疗结束后改用含拓扑替康的方案维持/巩固（表 2）。局限期患者21例（72.4%），广泛期8例（2 7.6 %）。一线化疗共4个疗程，其中2 8例（9 6.4 %）一线方案同时包含了足叶乙甙和铂类，一线治疗有效率为8 9. 6 %。拓扑替康维持/巩固单药9例（31.0%），与顺铂联合20例（69.0%）。中位化疗周期数为2。治疗后CR占27.6%（8/29），PR占24.1%（7/29），SD占17.2%（5/29），PD占10.3%（3/29），6例患者缺乏疗效评价资料（6/29, 20.7%）。

### 2.4 生存分析

全组患者进行统计分析时仅3名存活，MST为20个月（95%CI: 15.9-24.1），1年、2年及5年生存率分别为88%、53%和13%。其中局限期患者MST为24个月（95%CI: 16.0-32.0），1年、2年及5年生存率分别为94%、62%和21%；广泛期MST为19个月（95%CI: 16.4-21.6），1年、2年及5年生存率分别为77%、36%和0。拓扑替康挽救治疗组M S T为2 7个月（9 5 % C I : 17.3-36.6），1年、2年及5年生存率分别为91%、62%和5%。而维持/巩固治疗组患者MST为20个月（95%CI:12.2-27.8），1年、2年及5年生存率分别为86%、47%和19%。*Log-rank*法分析显示两组患者MST无统计学差异（*P*=0.89）（图 1）。*Cox*回归法对年龄（≥65岁或 < 65岁）、分期、PS评分、碱性磷酸酶（升高或正常值上限以下）、白蛋白（正常值下限以下或正常）、拓扑替康使用阶段（维持/巩固或挽救治疗）进行多因素分析显示，仅性别（男性，OR=2.55，95%CI：1.02-6.36）和分期（广泛期，OR=2.01，95%CI：1.04-3.90）是影响预后的独立危险因素。

**1:**
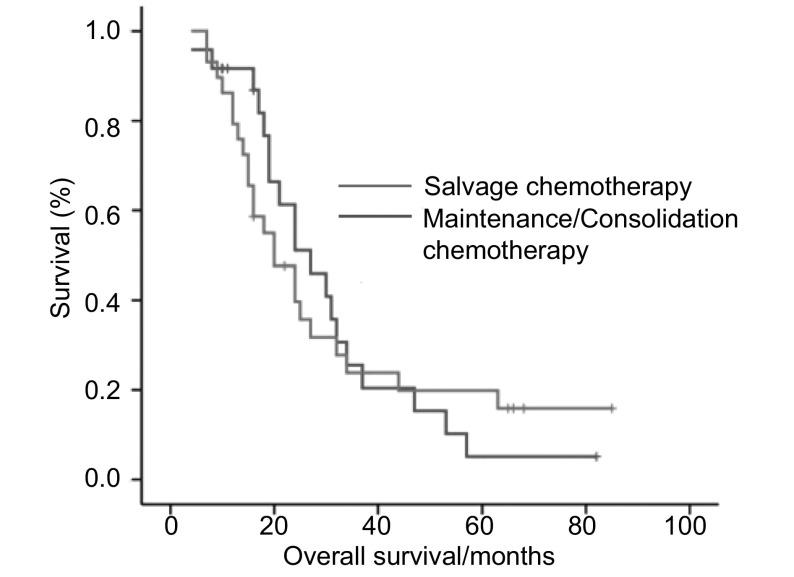
拓扑替康维持/巩固组和挽救治疗组总生存的*Log- rank*检验（*P*=0.89） *Log-rank* comparison of overall survival of SCLC patients receiving topotecan as salvage and maintenance/ consolidation treatment (*P*=0.89)

### 2.5 毒副反应

本研究显示拓扑替康最主要的毒副反应是骨髓抑制（表 3）。拓扑替康挽救化疗组可评价毒副反应的患者24例，其中3例（12.5%）出现Ⅲ-Ⅳ级白细胞降低，9例（37.5%）出现Ⅲ级血小板降低，没有Ⅳ级血小板降低，6例（25.0%）出现Ⅲ-Ⅳ级的贫血。接受拓扑替康维持/巩固化疗可评价毒副反应的患者29例，其中6例（20.7%）出现Ⅲ-Ⅳ级白细胞降低，6例（20.7%）出现Ⅲ级血小板降低，没有Ⅳ级血小板降低，3例（10.3%）出现Ⅲ- Ⅳ级贫血，3例（10.3%）出现Ⅲ度胃肠道反应。其他非血液学毒性如脱发、眩晕、乏力等都很轻微并且可以耐受。

**3:** 按NCI CTCAE 3.0版的拓扑替康维持/巩固及挽救治疗的3/4级毒性 Grade 3/4 toxicities of topotecan in maintenance/consolidation and salvage chemotherapy

Toxicities	Maintenance/Consolidation chemotherapy (*n*=29)	Salvage chemotherapy (*n*=24)	*P*
Hematologic toxicities			
Leukopenia			0.48
Grade 3	4(13.8%)	2 (8.3%)	
Grade 4	2 (6.9%)	1 (4.2%)	
Thrombocytopenia			0.23
Grade 3	6 (20.7%)	9 (37.5%)	
Grade 4	0	0	
Anemia			0.27
Grade 3	1 (3.4%)	5 (20.8%)	
Grade 4	2 (6.9%)	1 (4.2%)	
Nonhematologic toxicities (Grade 3/4)			
Nausea	3 (10.3%)	0	0.24
Vomiting	3 (10.3%)	0	0.24
Anorexia	4(13.8%)	0	0.12
Diarrhea	1 (3.4%)	1 (4.1%)	0.89
Alopecia	2 (6.8%)	2 (8.3%)	0.65

## 3 讨论

SCLC二线治疗有效率较一线治疗明显降低。耐药复发患者对大部分二线化疗方案有效率约为10%或更低；而敏感复发患者挽救治疗的有效率约为25%。复发进展后的患者中位生存时间在4个月-5个月，预后很差。拓扑替康单药是目前治疗敏感复发SCLC的标准方案。

本研究进行拓扑替康挽救化疗的患者中，41.7%为耐药复发，且37.5%的患者为三线或三线以后的治疗，但仍有较高的有效率（41.7%），较既往报道的稍高，可能与部分患者接受了拓扑替康与顺铂联合化疗，较单药拓扑替康提高了有效率。

我们的回顾性研究将拓扑替康提前用于一线化疗后获得CR、PR或SD的SCLC患者，观察能否提高化疗有效率、延长肿瘤缓解时间并改善总生存。结果显示，一线治疗取得肿瘤控制的患者在化放疗结束后改用含拓扑替康方案维持/巩固，提高了有效率，一线化疗达CR的患者有3例，维持/巩固治疗结束后，CR的患者增至8例（27.6%），还有7例（24.1%）在维持/巩固阶段仍能继续PR。经我们治疗的SCLC疗效与生存较既往报道的稍好，局限期MST为24个月，1年、2年及5年的生存率分别为94%、62%和21%；广泛期MST为19个月，1年、2年及5年的生存率分别为77%、36%和0。研究中挽救治疗组和维持/巩固组患者各项临床特征都是均衡的。无论单因素还是多因素分析，维持/巩固治疗并不能延长SCLC患者的生存，这与E7593试验结果一致。E7593试验结果显示巩固/维持组仍有2%的患者取得CR，5%的患者达PR，PFS较观察组稍有延长，但也未见生存优势。但该研究全部为广泛期SCLC，而我们的研究在维持/巩固组包含了72.4%的局限期患者，且69%的患者接受的是拓扑替康与顺铂联合化疗，所以可能比该研究的有效率高。该试验的研究者分析认为，拓扑替康维持/巩固治疗在该研究中未见OS延长，可能因为观察组患者在疾病进展后也接受了拓扑替康治疗，这导致治疗组的PFS延长不能转化为生存优势，也可能受治疗组后续治疗不确定性的影响。我们的研究证实，即使在后续治疗中使用拓扑替康，也并没有显示出与拓扑替康维持/巩固治疗的生存差异。作者分析导致这一阴性结果的另一个原因可能与EP方案和拓扑替康的给药顺序有关：研究设想4个疗程含足叶乙甙方案后给与拓扑替康维持/巩固，可抑制足叶乙甙这一拓扑异构酶Ⅱ抑制剂导致的拓扑异构酶Ⅰ上调，从而给患者带来临床获益。但正好相反，其他一些临床前研究^[9]^显示拓扑替康在足叶乙甙前给药有最大协同作用。目前文献报道^[10]^拓扑替康与顺铂联合一线治疗广泛期SCLC，无论有效率还是总生存均与标准EP方案相当。因此，是否可将拓扑替康用于SCLC一线治疗，用足叶乙甙维持/巩固，值得进一步研究。本次研究病例数较少也可能导致了阴性的研究结果。

本次研究显示，拓扑替康有较高的致白细胞和血小板下降的骨髓毒性，但将其提前用于维持/巩固治疗，骨髓毒性并没有较挽救化疗明显增加，并且拓扑替康的骨髓毒性是可预期的，无累积性。此外拓扑替康非血液学毒性轻微，化疗耐受性较好。

由于本研究是回顾性的，且纳入病例数较少，结果可能存在偏倚。纳入分析的患者局限期占2/3，比既往报道的比例高，可能与诊断当时头颅MRI、腹部CT和全身骨显像并不是常规检查手段，有些患者转移灶未检出而被诊断为局限期有关。

综上所述，作为推荐的SCLC的二线用药，将拓扑替康提前用于一线取得缓解或稳定后的维持/巩固治疗能提高一线治疗的有效率，但在我们的研究中并没能转化成生存优势，将拓扑替康用于SCLC的维持/巩固治疗值得进一步研究。
